# Adjunctive Therapy of Text4Support for Treatment-Resistant Depression Patients Receiving Repetitive Transcranial Magnetic Stimulation. A Multicenter Randomized Controlled Pilot Trial

**DOI:** 10.1192/j.eurpsy.2023.1774

**Published:** 2023-07-19

**Authors:** M. K. Adu, R. da Luz Dias, B. Agyapong, M. Flynn, S. Hassan, S. Sridharan, M. K. White, V. Agyapong

**Affiliations:** 1 Psychiatry; 2 Dalhousie University, Halifax NS, Halifax; 3 Psychiatry, University of Alberta, Edmonton, Canada

## Abstract

**Introduction:**

Despite several treatment strategies for treatment-resistant depression (TRD) exist, including the use of repetitive transcranial magnetic stimulation (rTMS), new therapeutic options are being introduced. Text4Support is a form of cognitive behavior therapy that allows patients with depression to receive daily supportive text messages that seek to correct or alter negative thought patterns through positive reinforcement. Text4Support is deemed a useful augmentation treatment strategy for patients with TRD. It is however currently unknown if adding the Text4Support intervention will enhance patients with TRD’s response to rTMS treatments

**Objectives:**

This study aims to assess the initial comparative clinical effectiveness of rTMS when used with and without the Text4Support program as an innovative patient-centered intervention for the management of participants diagnosed with TRD.

**Methods:**

This study is a multicentered prospective, parallel-design, two-arm, rater-blinded randomized controlled pilot trial. In total, 200 participants diagnosed with TRD will be randomized to one of two treatment arms (rTMS alone and rTMS with Text4Support). Participants in each arm will be made to complete evaluation measures at baseline, 1,3, and 6 months. The primary outcome measure will be the mean change to scores on the Hamilton Depression Rating Scale. Patient service utilization data and clinician-rated measures will also be used to gauge patient progress. Patient data will be analyzed with descriptive statistics, repeated measures, and correlational analyses.

**Results:**

The result of the study is expected to be available 18 months after the start of recruitment. We hypothesize that participants enrolled in the rTMS plus Text4Support intervention will achieve superior outcomes compared with participants enrolled in the rTMS treatment alone.

**Conclusions:**

The concomitant application of the combination of these two treatment techniques has not been investigated previously. Therefore, we hope that this project will provide a concrete base of data to evaluate the practical application and efficacy of using a novel combination of these two treatment modalities.

**Disclosure of Interest:**

None Declared

